# Surgical complications in zygomatic implants: A systematic review

**DOI:** 10.4317/medoral.21357

**Published:** 2016-10-01

**Authors:** Pedro Molinero-Mourelle, Laura Baca-Gonzalez, Baoluo Gao, Luis-Miguel Saez-Alcaide, Alexandra Helm, Juan Lopez-Quiles

**Affiliations:** 1 DDS, Postgraduate student. Department of Buccofacial Prosthetics, Faculty of Dentistry, Complutense University of Madrid; 2 UGRD, Undergraduate student. Faculty of Dentistry, Complutense University of Madrid; 3 DDS, Postgraduate student. Department of Medicine and Oral Surgery, Faculty of Dentistry, Complutense University of Madrid; 4 DDS, MD, PhD, Associate Professor. Department of Medicine and Oral Surgery, Faculty of Dentistry, Complutense University of Madrid

## Abstract

**Background:**

The use of zygomatic implants in the prosthetic rehabilitation of the patient with severe maxillary bone atrophy is another therapeutic alternative, not exempt from complications. The main objective of this review is to analyze and describe the most frequent surgical complications associated with the use of zygomatic implants.

**Material and Methods:**

An electronic database search on PubMed, along with a manual search, without taking into account date nor language, was undertaken by two observers, selecting studies that comprised a study period from 6 to 12 months, any type of clinical trial, and series that included a follow-up and/or review period during the aforementioned margin, that mentioned at least two types of complications.

**Results:**

Out of the initial search that yielded 455 studies, 67 were considered potentially relevant for the present study, out of which 14 were finally selected. Out of the most frequent surgical complications, sinusitis (3,9%) and failure in osseointegration (2,44%) are highlighted.

**Conclusions:**

The analysis of the results shows that the most frequent complications are sinusitis and failure in osseointegration of the zygomatic implant. However, a standardised data collection system for the data on complications is needed.

**Key words:**Implant, zygomatic implant, surgery, complications.

## Introduction

The presence of inadequate bone quantity as seen in patients with atrophied maxillae poses a problem for implant placement, implying various bone augmentation procedures such as block bone grafting or sinus floor elevation, which, in both cases, may imply multiple interventions. On the other hand, the zygomatic implant technique results less invasive and more predictable (1-4).

The zygomatic implant was originally developed by Brånemark in 1989, for the rehabilitation of atrophied maxillae in cancer patients that had undergone partial or total maxillectomy. Currently, zygomatic implants are mainly indicated for dental rehabilitation in atrophic maxillae. An implant with the following characteristics was designed: a 45-degree-angled head, a diameter of 4.5 mm at its widest part, and a length of 30 to 50 mm. The implant follows an insertion path from the palatal aspect of the alveolar process, following the zygomatic alveolar crest until its anchorage in the malar body, which constitutes an excellent buttress due to its great bone density (5).

However, given that the use of the zygomatic implant is a surgical intervention in nature, there are numerous studies that mention that its use is not exempt from complications (6).

The main objective of the present article is to carry out a systematic review on the cases and studies in the literature in order to establish the most frequent surgical complications associated with treatment using zygomatic implants.

## Material and Methods

An electronic search was undertaken in December 2015 in the PubMed database (U.S. National Library of Medicine, National Institute of Health). The search strategy was {Subject AND Adjective} {Subject: (zygomatic OR zygoma OR zygomaticus [Title]) AND Adjective: (implant OR implants OR fixture OR fixtures [Title])}, without taking into account neither language nor date. Two observers examined the resulting articles in order to discern which complied with inclusion criteria, based on their title and abstract. In the event that both observers did not agree upon evaluation, a third observer undertook the final assessment.

The following inclusion criteria were used in the present study 1) series of patients with severe maxillary atrophy, that had been treated with zygomatic implants; 2) such series had to include direct clinical data; 3) any type of study was valid (habitual clinical practice, clinical trials, observational studies); 4) only previous reviews were included if they provided data; 5) the patients included in the series had to have been monitored with a post-surgical review for a minimum period of 6 to 12 months; 6) the clinical series (clinical trials and studies) had to include direct data of at least two types of surgical complications arisen by the use of zygomatic implants of the mentioned in the present review, (non-osseointegrated implants, bruising, sinusitis, fistulae at implant level, paresthesia, labial laceration and/or local infections). Due to the lack of literature reviews, the number of categories was reduced from two to one, in order to include monographic systematic reviews on any type of complication.

Exclusion criteria used in the review were: 1) series that did not provide clinical data; 2) individual case reports; 3) reviews that did not include data or specific references to studies; 4) series of patients that did not include a follow-up period and/or review during the aforementioned period; 5) published series that did not include direct data of at least two types of surgical complications. References used in excluded articles were also included during the analysis in the present review.

## Results

**Figure 1:**
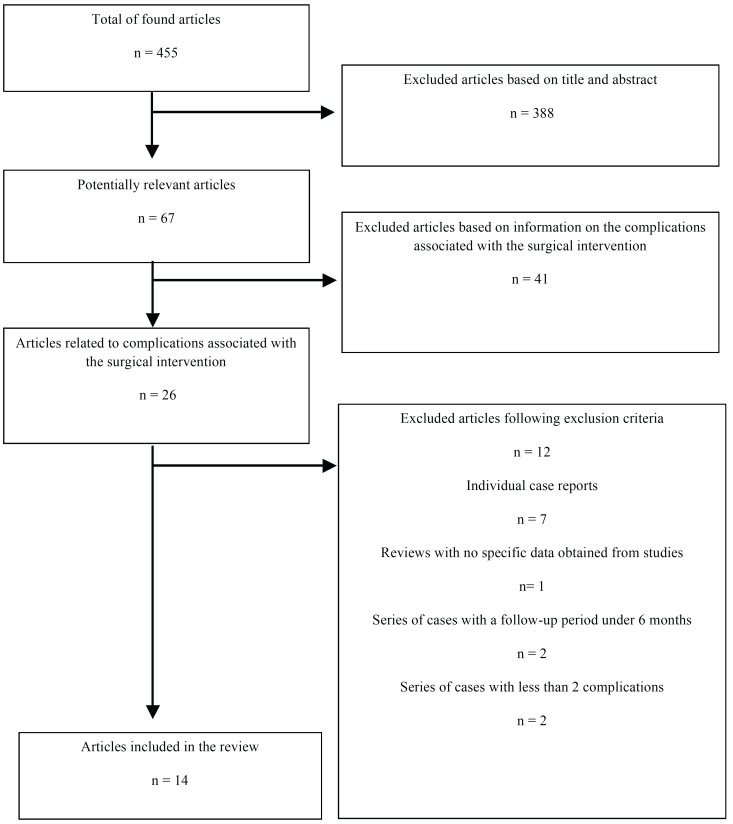
Flow diagram.

The initial search yielded a total of 455 studies (Fig. 1). 67 were considered potentially relevant, based on their title and abstract. Following their lecture, 26 articles were included, out of which 12 were excluded, as they did not comply with the inclusion criteria: 7 individual case reports, 1 review that did not include specific study data, 2 reviews that did not monitor patients for a minimum follow-up period of 6 to 12 months and 2 series of cases with no direct data on at least two types of surgical complications. Finally, 14 articles were selected for the present review ( Table 1), and their respective surgical complications highlighted ( Table 2).

**Table 1:**
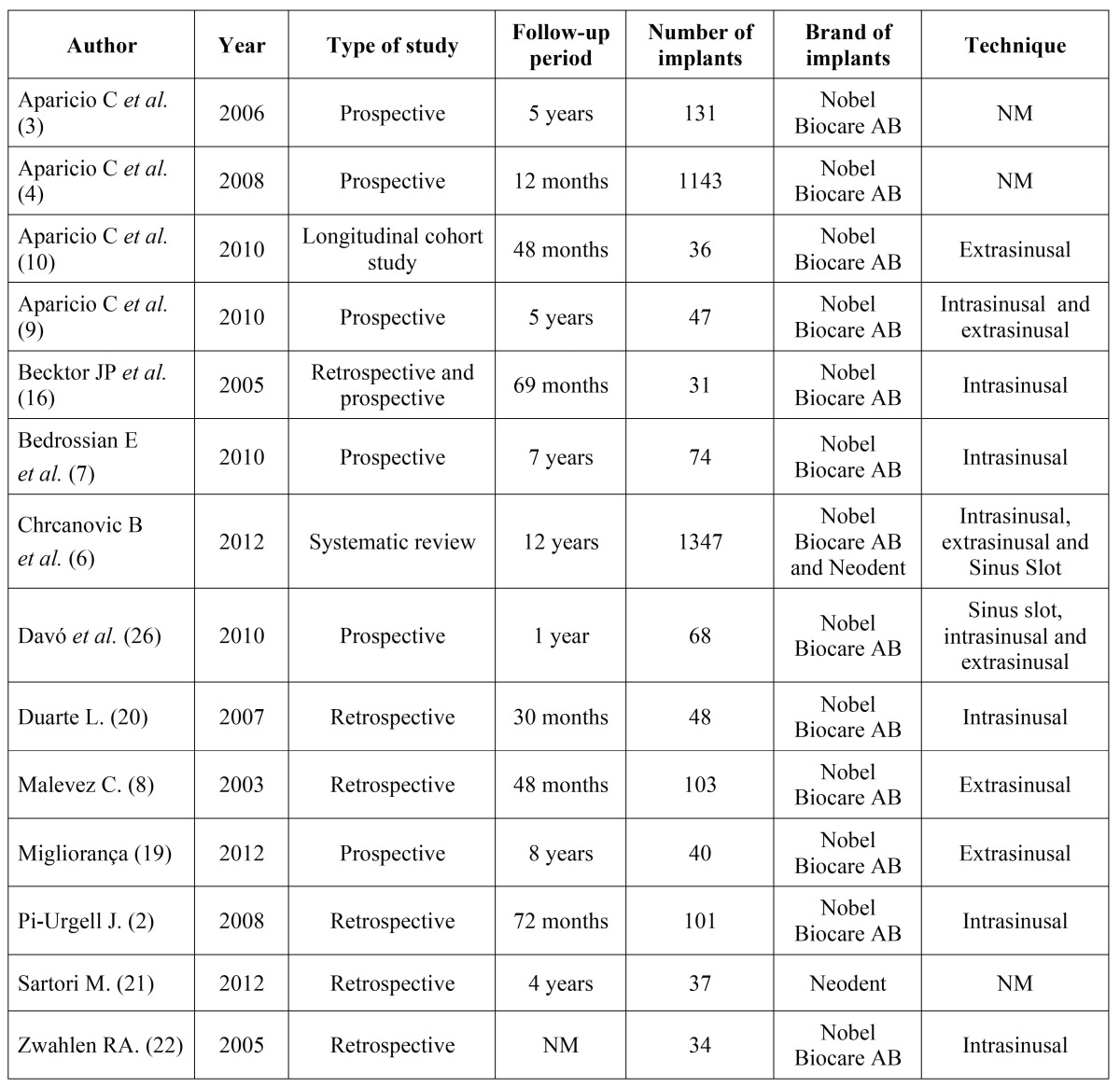
Studies that mention surgical complications associated with zygomatic implants.

**Table 2:**
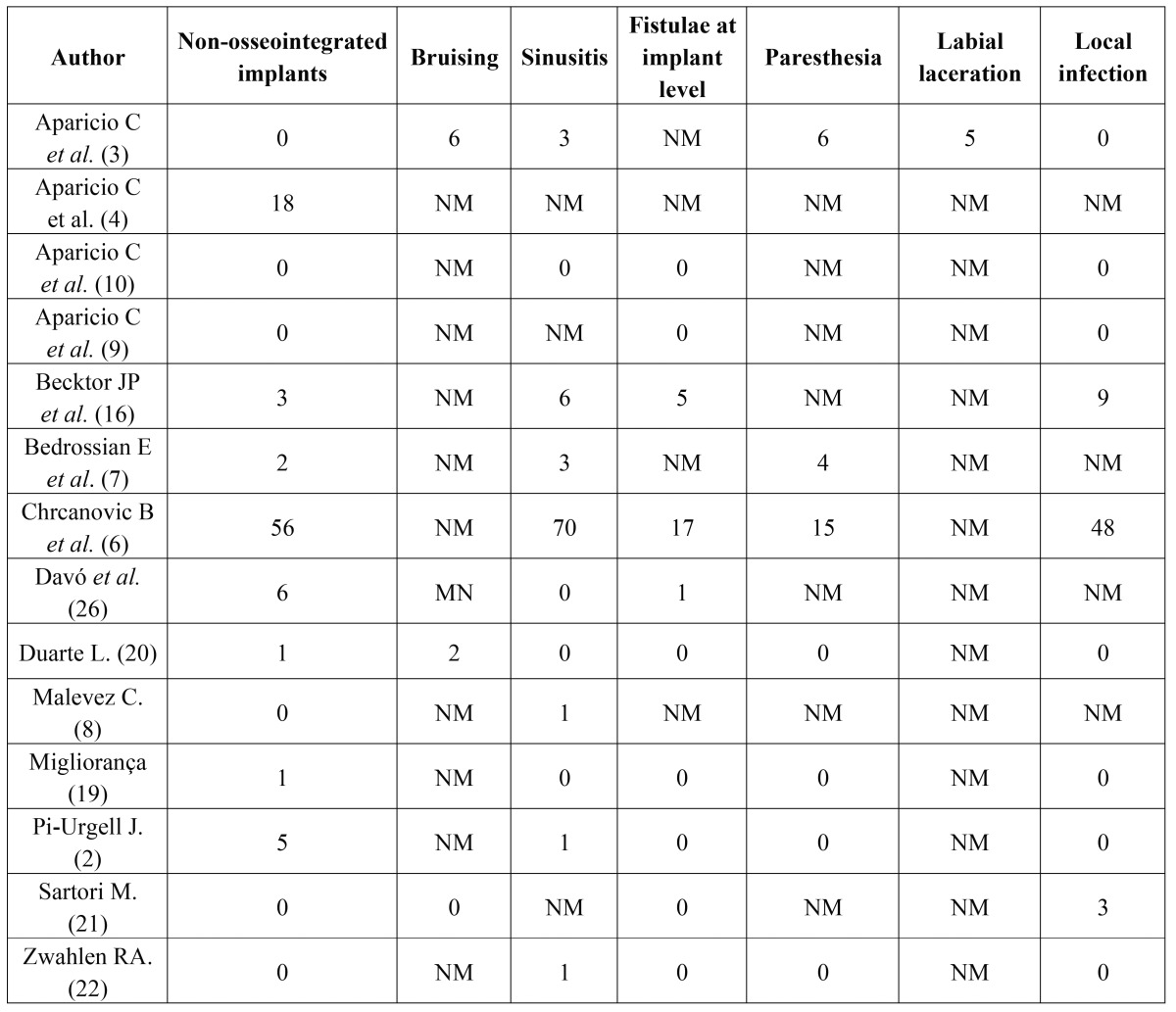
Surgical complications associated with zygomatic implants.

## Discussion

The present review includes a series of limitations, mentioned below:

- Variability in the type of study, in which clinical trials, prospective and retrospective patient cohorts and longitudinal studies were included.

- Variability in inclusion and exclusion criteria in each of the published series.

- Unlike other treatments, there is no normalised scale that registers or scales the intensity of the postsurgical complications that arise in this type of intervention.

- Each published series has compiled the complications pertaining the patients that form it, without a standardization in data collection. In the same manner, only the number of implants can be known; in many cases, the number of patients that compose the series can not be determined.

Compared to the conventional implant, the biomechanical situation of the zygomatic implant varies. The zygomatic implant is much longer, the main anchorage is located far away from the loading point and it is positioned in an angled manner, which results in an unfavourable biomechanical situation when they are considered in an isolated manner. The trabecular structure of the zygomatic bone, not so adequate for implant support, is compensated due to the stability provided by the maxillary sinus cortical bone located at the crestal section of the implant. Therefore, rehabilitation must be conceived as a unique piece, composed by a rigid bar, which includes from two to four conventional implants located in the anterior maxilla (3,7).

Consequently, sufficient bone quantity is necessary in the anterior region, as well as other bone augmentation procedures (4). Alternatively, two zygomatic implants at either side can be placed, a treatment approach known as Quad Zygoma. In other treatment approaches, zygomatic implants are combined with pterygoid or tuberosity implants. They are also indicated in partially edentulous patients (8-12).

Treatment options depend on the surgical technique, where either sedation, or preferably, general anaesthesia, are used. The intrasinus technique originally described by Brånemark involves the opening of a sinus window, the reflection of the Schneiderian membrane, and the placement of the implant from the bone crest up to the malar bone, through the maxillary sinus, protecting the membrane’s integrity. Posteriorly, Stella and Warner developed the sinus slot technique (13), which requires the opening of a small slot or window without taking into account the integrity of the Schneiderian membrane, in order to orientate the implant and improve the visibility of the malar body. Lastly, the extrasinus technique places the implant from outside the sinus towards its anchorage in the zygomatic bone, up to the alveolar crest.

Although the present approach is the extrasinus technique, the selection of one procedure or another, as well as the possible complications, depend on the patient’s anatomic biotype.

1. Sinusitis

The zygomatic implant placement may result in a foreign body reaction (14,15), in the form of inflammation of the sinus membrane, may be triggered by a treated implant surface against a finished one, an oroantral communication produced by perforation of the Schneiderian membrane, and a lack of osseointegration of the coronal part of the implant (16).

In the majority of the revised studies, sinusitis is the most frequently observed complication, with an average prevalence of 3,9 zygomatic implants out of every 100 placed. Other authors also consider this as the most relevant complication, such as Becktor *et al.*, with 19,4% cases (16) and Chrcanovic *et al.* with 5,2% (6). Great discrepancies in the results obtained by Becktor may be due to, according to the author, difficulty in maintaining optimum hygiene at the posterior palatal emergency; transversal mobility produced by functional forces when there is a lack of osseointegration and bone-implant contact at a marginal level; and the internal design of the implant, which may produce an oroantral communication. However, the extrasinus technique permits a more favourable emergence of the implant, and facilitates adequate hygiene maintenance of the area (3,10). As for the design of the implant, some authors mention that in later studies, reported rates on this complication are not as high (4,11), therefore, more conclusive studies in this area are needed. Another relevant fact that must be taken into account is the presence of sinusitis prior to the surgery (17).

2. Non-osseointegrated implants

Causes related to the lack of osseointegration include overheating, contamination and trauma during the surgery, insufficient bone quantity or quality, lack of primary stability and incorrectly indicated immediate loading (18).

Non-osseointegrated implants appear with a mean frequency of 2,44%, where authors such as Becktor *et al.* with 9,7% (16) and Chrcanovic *et al.* with 4,2% (6) and Migliorança *et al.* with 2,5% (19) can be mentioned. Below the aforementioned average, other authors can be noted, such as Duarte *et al.* with 2,08% (20), Aparicio *et al.* with 1,5% (4) and Migliorança *et al.* with 2,5% (19); others such as Sartori *et al.* (21) and Zwahlen *et al.* (22) report an osseointegration success rate of 100%. In the long term, studies report that survival rate of zygomatic implants is comparable to that of conventional implants (6).

3. Local infections

Local infections or mucositis are directly related to the appearance of sinusitis, favoured by the lack of osseointegration, lack of contact between the implant and the bone crest, superficial infection and lack of cicatrisation of the soft tissues. Prosthodontic rehabilitation also plays a relevant role (23).

The prevalence obtained in the present study is of 4%, coinciding with the result obtained by Chrcanovic, who shows a similar result of 3,6%, also being the third most frequent complication for the author.

4. Fistula at implant level

Lack of osseointegration at the marginal area of the implant at its palatal aspect, along with functional forces, may increase the risk of oroantral communication and the posterior development of sinusitis (24,25).

In a systematic review of 42 articles, 17 cases of oroantral fistulae were observed, quoted by six authors. (7) The frequency of this complication in the mentioned studies varies between 1,5 and 7,5% (6), except in the case of Becktor *et al.*, who reached 29% (16).

The present study found a frequency of 2%, similar to the one obtained by Davó, who obtained a result of 1% (26). Some studies highlight that the use of the definitive pillar and immediate loading, from the start, probably reduce the possibilities of oroantral communications (9).

5. Paresthesia

In a systematic review conducted by Chrcanovic *et al.*, 15 cases of paresthesia from affection of infraorbitary and zygomaticofacial nerves were reported (6), however, in the majority of reviewed cases, paresthesia remits between 3 and 8 weeks postintervention. (3,7) 

Paresthesia has a frecuency of 1,36%. For Bedrossian (7) and Aparicio (9), paresthesia is considered as the most frequent complication, with a prevalence of 5,4% and 4,6%, respectively. The incidence can vary, being a complication closely linked to the surgeon’s expertise and the discipline of the surgical team.

6. Bruising

It ranks fourth place in terms of frequency, with 3,9% (3,20). The incidence is probably higher, due to the fact that many authors do not mention this as a complication, possibly due to its less alarming clinical manifestations, being self-limited, and associated with the postoperative period. Lastly, it would not be a complication exclusively linked to rehabilitation with zygomatic implants.

7. Labial laceration

Possibly one of the most common complications, and as mentioned in the case before, it is also underdocumented, only being mentioned by Aparicio *et al.* (4).

Lastly, some studies mention not having any complications in any of their interventions using zygomatic implants (9,10).

## Conclusions

It is possible to conclude that rehabilitation using zygomatic implants is a consolidated therapeutic option, and although it is a predictable technique, it does not lack in possible complications, therefore, it should be reserved only to professionals with vast surgical experience, as it requires a long learning curve and prior experience with conventional implants.

Based on the revised studies, the most frequent complications are sinusitis and lack of osseointegration, however, their treatment and control are consolidated in standard clinical practice.

More studies need to be developed, being necessary a normalised and standardised data registration system for its posterior analysis.
